# Treatment Outcomes of Excisional Versus Conservative Surgery for Endogenous Cesarean Scar Pregnancy: A Retrospective Cohort Study

**DOI:** 10.1155/ogi/5246199

**Published:** 2026-09-16

**Authors:** Zhujie Wang, Xingmiao Li, Feng Qi, Jianwei Zhou, Ning Wang, Meijun Guo, Mengjia Yu, Yanan Yang, Xuelu Zhu, Xiuxiu Jiang

**Affiliations:** ^1^ Department of Family Planning, Women’s Hospital, Zhejiang University School of Medicine, Hangzhou City Zhejiang, 310006, China, zju.edu.cn; ^2^ Department of Gynecology, Taizhou Hospital of Zhejiang Province, Zhejiang University, Taizhou City Zhejiang, 317000, China, zju.edu.cn; ^3^ Department of Gynecology, The Second Affiliated Hospital, Zhejiang University School of Medicine, Hangzhou City Zhejiang, 310006, China, zju.edu.cn; ^4^ Department of Biostatistics, Clinical Research Institute, Institute of Advanced Clinical Medicine, Peking University, Beijing 100871, China, pku.edu.cn; ^5^ Zhejiang Provincial Clinical Research Center for Obstetrics and Gynecology, Zhejiang Key Laboratory of Maternal and Infant Health, Department of Family Planning, Women’s Hospital, Zhejiang University School of Medicine, Hangzhou City Zhejiang, 310006, China, zju.edu.cn

**Keywords:** cesarean scar diverticulum, CSP classification, residual myometrium thickness

## Abstract

**Objective:**

To compare treatment outcomes (including cesarean scar diverticulum and residual myometrium thickness [RMT]), among patients with endogenous cesarean scar pregnancy (CSP) stratified by the Chinese classification.

**Methods:**

.

**Study design:**

A 6‐year multicenter retrospective observational cohort study of CSP patients in China.

**Participants:**

A total of 940 women with endogenous CSP underwent vacuum aspiration surgery (VAS), hysteroscopic conservative surgery (HCS), and excisional surgery (ES).

**Main Outcome Measures:**

Detection rate of cesarean scar diverticulum and preoperative–postoperative differences in RMT.

**Results:**

The postoperative detection rate of cesarean scar diverticulum was 77.5% (555/716) in the Type 2 CSP group, significantly higher than that of 64.3% (144/224) in the Type 1 CSP group (*p* < 0.001). The preoperative–postoperative change in RMT was 0.13 ± 0.11 for Type 2 CSP patients, significantly higher compared with −0.10 ± 0.16 in Type 1 CSP patients (*p* < 0.001).

**Conclusion:**

ES helps lower the incidence of cesarean scar diverticulum and improve RMT.

## 1. Introduction

Cesarean scar pregnancy (CSP) refers to a condition in which the gestational sac is partially or completely embedded in the niche or in the scar from a previous cesarean section and is surrounded by myometrium and fibrous tissue [1, 2]. The incidence of CSP is approximately 1:2000, but the incidence is increasing because of increasing cesarean section rates worldwide and more widespread use of transvaginal color Doppler sonography as a diagnostic method [1, 3]. Termination of pregnancy is often recommended once CSP is diagnosed. Delayed diagnosis or treatment may put the patients at high risk of massive, life‐threatening hemorrhage, placenta accreta, or even uterine rupture because of the poor contractility of the lower segment myometrium and the thin myometrium in the scar [4, 5]. Clinically, common options for CSP treatment include the use of methotrexate (MTX) via different routes, hysteroscopy, vacuum aspiration, laparoscopy, or laparotomy to repair the CSP site, and, when necessary, uterine artery embolization or uterine artery ligation may be needed [4, 6–9]. Compared with surgical treatment, medical treatment is not typically recommended as a first‐line option by clinicians because of its lower success rate and associations with a slower decline in β‐hCG levels and longer hospitalization duration [10–12]. Cesarean scar diverticulum represents a burgeoning complication associated with cesarean sections. With the increasing prevalence of cesarean deliveries, the iatrogenic complications linked to this procedure are increasing. Among these complications are placenta accreta, scar ectopic pregnancy, and the relatively newly characterized uterine niche, which has been increasingly discussed in recent medical literature. In addition to uterine isthmocele, cesarean scar diverticulum arises as an iatrogenic defect in the myometrium at the site of a prior cesarean scar, resulting from inadequate tissue healing [13]. The laparoscopic resection of a large uterine niche can significantly alleviate patients’ symptoms and increase the residual myometrium thickness (RMT) [14]. However, the effect of CSP repair surgery on cesarean scar diverticulum and the RMT remains unclear.

CSP was classified into endogenous and exogenous types according to its growth pattern and the implantation depth of the fertilized egg at the scar site, which was also known as the international classification for CSP [15–17]. Endogenous CSP occurs due to the implantation of the amniotic sac on a scar with progression of the pregnancy in the cervico‐isthmic space and in the uterine cavity. Exogenous CSP occurs due to deep implantation of the amniotic sac in a cesarean scar defect, with progression toward rupture and bleeding during the first trimester of pregnancy [15, 17]. The occurrence of CSP is associated with the formation of a cesarean scar diverticulum after a cesarean section. However, reports on the effect of different treatment modalities on cesarean scar diverticulum following CSP treatment, as well as on changes in RMT, are lacking. In 2016, considering the relationship between the embryo sac and scar tissue and the RMT, the Obstetrics and Gynecology Branch of the Chinese Medical Association reached a consensus that the scar thickness is a standalone high‐risk factor and an important indicator for guiding the diagnosis and treatment of CSP [18]. CSPs are classified as Types 1, 2, and 3 according to the relationship between the embryo sac and scar tissue and RMT. A CSP with an RMT > 3 mm is defined as a Type 1 CSP, and a CSP with an RMT < 3 mm is defined as a Type 2 CSP. In Type 3 CSP, the gestational sac bulged out of the cesarean scar or formed an amorphous mass with rich vascularity at the cesarean scar and with an RMT < 3 mm. This Chinese classification separates Type 2 CSP from endogenous CSP based on the criterion that the RMT is < 3 mm. Therefore, endogenous CSP in the international classification is composed of Type 1 CSP and Type 2 CSP in the Chinese classification. Exogenous CSP is consistent with Type 3 CSP.

In our previously published retrospective study, we demonstrated that excisional surgery (ES) could improve subsequent pregnancy rates and reduce the recurrence risk of CSP compared with conservative surgery [19, 20]. In the present study, we adopted the same patient cohort (Ethics Approval Number: ZDFY2023‐4XPY101) to further compare the detection rate of cesarean scar diverticulum and preoperative–postoperative differences in RMT between ES and conservative surgery among patients with endogenous CSP.

## 2. Materials and Methods

### 2.1. Study Design

This was a retrospective cohort study in which endogenous CSP patients who underwent surgical treatment were recruited from three tertiary hospitals.

### 2.2. Study Duration

This study was conducted from January 1, 2019, to December 31, 2023. The follow‐up for pregnancy outcomes ended on January 1, 2025.

### 2.3. Study Setting

Endogenous CSP patients undergoing surgical treatment were recruited from the Women’s Hospital, School of Medicine, Zhejiang University (WHZJU), the Taizhou Hospital of Zhejiang Province (TZH), and the Second Affiliated Hospital of Zhejiang University School of Medicine (SAHZJU) from January 1, 2019, to December 31, 2023. All three hospitals involved in this study are large‐scale tertiary Grade A hospitals in China. Among them, WHZJU is the largest obstetrics and gynecology specialty hospital in Zhejiang Province, with an annual delivery volume exceeding 20,000 and a cesarean section rate of 40% over the past 6 years.

### 2.4. Study Population

A total of 1258 women with CSPs from three tertiary hospitals (WHZJU, TZH, and SAHZJU) were screened. Among them, 940 women with endogenous CSP who underwent vacuum aspiration surgery (VAS), hysteroscopic conservative surgery (HCS), and excisional resection (ES) for CSP were included in this study.

### 2.5. Selection Criteria

**FIGURE 1:**
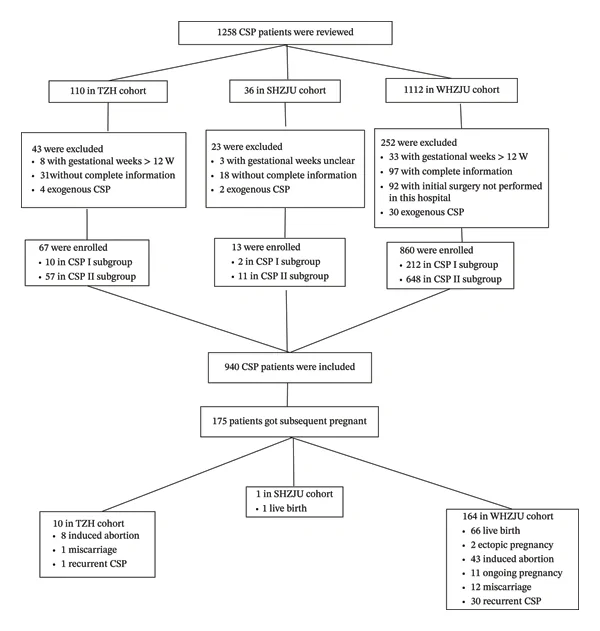
Flowchart of this retrospective observational cohort study conducted at three participating centers. TZH = Taizhou Hospital of Zhejiang Province. SAHZJU = Second Affiliated Hospital of Zhejiang University School of Medicine. WHZJU = Women’s Hospital, School of Medicine, Zhejiang University.

The clinical diagnosis of CSP relied mainly on clinical manifestations, the serum β‐HCG level, and 3D ultrasound and was ultimately confirmed by pathological diagnosis. The inclusion criteria were as follows: Endogenous CSP patients who underwent surgical treatment were recruited from these three hospitals. Individuals who met any of the following criteria were excluded: those with incomplete clinical data or follow‐up data; those whose initial surgeries performed outside the three participating hospitals; and those with exogenous CSP (Type 3 CSP). (Figure 1).

### 2.6. Study Tools

The study tools included medical records and telephone follow‐up communications. Data were obtained by querying the medical records of the three tertiary hospitals and conducting telephone follow‐up communications.

### 2.7. Study Procedure

This was a retrospective cohort study in which endogenous CSP patients who underwent surgical treatment were recruited from the three study hospitals from January 1, 2019, to December 31, 2023. Ethics approval for this study was obtained from the three hospitals (WHZJU: IRB‐20240142‐R; TZH: K20190117; and SAHZJU: 2024‐0304). All participants provided written consent. Data were obtained by querying the medical records of the three tertiary hospitals and conducting telephone follow‐up communications. All included data were consolidated and analyzed by personnel at WHZJU.

### 2.8. Operation Definitions

ES included excision of the CSP and closure of the uterine incision through a transvaginal approach or a hysteroscopy‐assisted laparoscopic approach. Conservative surgery included VAS and HCS. The treatment choices were as follows: VAS for Type 1 CSP; HCS or ES for Type 2 CSP. For patients with extremely rich blood flow at the implantation site of the CSP or those with a very thin or discontinuous RMT, uterine artery embolization was performed. Detailed descriptions of the operative procedures for VAS, HCS, and ES are provided in our previous study [19, 20]. A multidisciplinary team comprising surgeons, radiologists, and patients collaboratively determined the operative strategy. The clinical decision was informed by three principal categories: patient‐related determinants (age, reproductive intention, prior surgical history, and baseline functional status), disease‐specific parameters (CSP type, RMT, and estimated risk of hemorrhage), and the surgeon’s technical proficiency. Selection bias was minimized through the combination of propensity score matching (Supporting information [available here]) and stratified analysis.

### 2.9. Study Variables

The primary outcomes included the detection rate of cesarean scar diverticulum and preoperative–postoperative differences in RMT, while the secondary outcomes included surgical complications. Cesarean scar diverticulum was defined by 3D ultrasound as a fluid‐filled anechoic space in the anterior isthmus connected to the uterine cavity. RMT was measured as the minimal residual myometrial thickness on 3D sonography. The preoperative–postoperative differences in RMT after surgery were defined as the change in RMT in the lower segment of the anterior isthmus before and after the surgery. Severe complications included uterine perforation, massive hemorrhage, hysterectomy, and blood transfusion.

### 2.10. Bias

The same ultrasound expert reviewed the ultrasound data to ensure that the diagnoses were consistent.

### 2.11. Data Analysis

Continuous variables are expressed as means ± standard deviations (SDs) or medians and interquartile ranges (IQRs). Categorical variables are expressed as frequencies (percentages). Between‐group comparisons of continuous variables were analyzed via independent *t* tests if the data were normally distributed or using the Mann‒Whitney *U* test, and those of categorical variables were analyzed via the *χ* test or Fisher’s exact *χ* test (depending on the characteristics of the data). The significance level was set at *α* = 0.05 (two‐sided). All analyses were conducted using R software Version 4.4.2 and SPSS Version 27.0 (IBM, Armonk, NY, USA).

## 3. Results

The baseline clinical characteristics of the 940 endogenous CSP patients in different groups are summarized in Table 1. Intergroup comparisons revealed no significant differences in these characteristics.

**TABLE 1:** Baseline characteristics of Type 1 CSP and Type 2 CSP patients.

Baseline characteristics^a^	Total *N* = 940	Type 1 CSP, 224 (%)	Type 2 CSP, 716 (%)	*p* value
Centers, *n* (%)				
WHZJU	860 (91.5)	212 (94.6)	648 (90.5)	0.153
TZH	67 (7.1)	10 (4.5)	57 (8.0)	
SHZJU	13 (1.4)	2 (0.9)	11 (1.5)	
Age, years	34.5 ± 4.7	34.7 ± 4.8	34.5 ± 4.6	0.563
Marriage, *n* (%)				
Married	886 (94.3)	213 (95.1)	673 (94.0)	0.539
Unmarried	54 (5.7)	11 (4.9)	43 (6.0)	
Smoking^b^, *n* (%)				
Yes	22 (2.3)	3 (1.3)	19 (2.7)	0.256
No	918 (97.7)	221 (98.7)	697 (97.3)	
Reproductive organ lesions, *n* (%)				
Yes	124 (13.2)	38 (17.0)	86 (12.0)	0.056
No	816 (86.8)	186 (83.0)	630 (88.0)	
BMI^c^, *n* (%)				
< 30 kg/m^2^	900 (95.7)	217 (96.9)	683 (95.4)	0.337
≥ 30 kg/m^2^	40 (4.3)	7 (3.1)	33 (4.6)	
Vaginal bleeding, *n* (%)				
Yes	599 (63.7)	148 (66.1)	451 (63.0)	0.402
No	341 (36.3)	76 (33.9)	265 (37.0)	
Abdominal pain, *n* (%)				
Yes	132 (14.0)	28 (12.5)	104 (14.5)	0.446
No	808 (86.0)	196 (87.5)	612 (85.5)	
Parity^d^, *n* (%)				
1	478 (50.9)	116 (51.8)	362 (50.6)	0.471
2	416 (44.3)	94 (42.0)	322 (45.0)	
≥ 3	46 (4.9)	14 (6.3)	32 (4.5)	
C‐section^e^, *n* (%)				
1	513 (54.6)	126 (56.3)	387 (54.1)	0.554
2	393 (41.8)	88 (39.3)	305 (42.6)	
≥ 3	34 (3.6)	10 (4.5)	24 (3.4)	
Gestational weeks, weeks	6.8 ± 1.4	6.7 ± 1.4	6.8 ± 1.4	0.329
Diameter of the pregnancy sac (cm)^f^	2.6 ± 1.3	2.2 ± 1.3	2.7 ± 1.3	< 0.001
Uterine artery embolization, *n* (%)				
Yes	98 (10.4)	5 (2.2)	93 (13.0)	< 0.001
No	842 (89.6)	219 (97.8)	623 (87.0)	

*Note:* This table was originally published as part of our previous work based on the same cohort study [19, 20]. Median (IQR): median and interquartile range.

Abbreviation: BMI, body mass index.

^a^Baseline characteristics are from the time of diagnosis of CSP.

^b^Smoking refers to smoking more than 10 cigarettes per day.

^c^ BMI is calculated as weight in kilograms divided by the height in meters squared.

^d^Parity refers to the number of times a woman has carried a pregnancy to full term and successfully delivered.

^e^C‐section refers to the number of cesarean sections a woman has undergone.

^f^Diameter of the pregnancy sac refers to the mean diameter of the pregnancy sac.

The mean diameter of the pregnancy sac in the Type 2 CSP group was 2.7 ± 1.3 cm, which was significantly higher than that in the Type 1 CSP group (2.2 ± 1.3 cm, (*p* < 0.001). The UAE usage rate in the Type 2 CSP group was 13.0%, which was significantly higher than that in the Type 1 CSP group (2.2%) (*p* < 0.001).

The detection rate of cesarean scar diverticulum in the Type 2 CSP group was 555 (77.5%), which was significantly higher than that in the Type 1 CSP group (144; 64.3%) (*p* < 0.001). The preoperative–postoperative difference in RMT in the Type 2 CSP group was 0.13 ± 0.11 cm, which was significantly higher than that in the Type 1 CSP group (−0.10 ± 0.16 cm) (*p* < 0.001). There were no significant differences in severe complications between the Type 1 CSP group and the Type 2 CSP group (Table 2).

**TABLE 2:** Comparison of the treatment outcomes of Type 1 CSP and Type 2 CSP patients.

Treatment outcome	Total *N* = 940	Type 1 CSP, 224 (%)	Type 2 CSP, 716 (%)	*p* value
Uterine diverticulum^a^, (%)				
Yes	699 (74.4)	144 (64.3)	555 (77.5)	< 0.001
No	241 (25.6)	80 (35.7)	161 (22.5)	
Value difference of RMT^b^ (cm)				
Means ± SDs	0.07 ± 0.16	−0.10 ± 0.16	0.13 ± 0.11	< 0.001
Severe complications^c^, (%)				
No	938 (99.8)	224 (100.0)	714 (99.7)	1.000
Yes	2 (0.2)	0 (0.0)	2 (0.3)	

*Note:* Uterine diverticulum, changes of residual myometrium thickness and severe complications were analyzed among all the CSP patients.

Abbreviations: CS, conservative surgery; ES, excisional surgery.

^a^Uterine diverticulum refers to the ultrasound diagnosis of a uterine diverticulum after CSP surgery.

^b^Value difference of RMT after surgery refers to the change in the thickness of the residual myometrium thickness in the lower segment anterior isthmus before and after the surgery.

^c^Severe complications include bleeding greater than 1000 mL, uterine perforation, or hysterectomy.

Given that different treatment modalities were performed for Type 2 CSP, the surgical approaches were further stratified. There were significant differences in the detection rate of cesarean scar diverticulum after surgery among the VAS, HCS, and ES subgroups (*p* < 0.001). There were significant preoperative intergroup differences in the RMT among the VAS, HCS, and ES subgroups (*p* < 0.001). (Table 3).

**TABLE 3:** Comparison of the treatment outcomes of different surgical procedures among Type 2 CSP patients.

Treatment outcome	VAS, 323 (%)	HCS, 359 (%)	ES, 34 (%)	*p* value
Uterine diverticulum after surgery, *n* (%)				
Yes	197 (61.0)	351 (97.8)	7 (20.6)	< 0.001
No	126 (39.0)	8 (2.2)	27 (79.4)	
Value difference of RMT (cm)				
Median (IQR)	0.11 (0.06–0.20)	0.10 (0.06–0.15)	0.30 (0.18–0.43)	< 0.001
Severe complications, *n* (%)				
Yes	2 (0.6)	0 (0.0)	0 (0.0)	0.295
No	321 (99.4)	359 (100.0)	34 (100.0)	
Uterine artery embolization, *n* (%)				
Yes	42 (13.0)	51 (14.2)	0 (0.0)	0.062
Yes	281 (87.0)	308 (85.8)	34 (100.0)	

Abbreviations: ES, excisional surgery; HCS, hysteroscopy conservative surgery; RMT, residual myometrium thickness; VAS, vacuum aspiration surgery.

The detection rate of cesarean scar diverticulum in the VAS subgroup was 197 (61.0%), which was significantly lower than that in the HCS subgroup 351 (97.8%) (*p* < 0.001) but significantly higher than that in the ES subgroup 7 (20.6%) (*p* < 0.001). The detection rate of cesarean scar diverticulum in the HCS subgroup was significantly higher than that in the ES subgroup (351 [97.8%] vs. 7 [20.6%], *p* < 0.001). The preoperative–postoperative difference in the RMT in the VAS subgroup was significantly higher than that in the HCS subgroup (0.11 [0.06–0.20] vs. 0.10 [0.06–0.15], *p* = 0.011) but significantly lower than that in the ES subgroup (0.11 [0.06–0.20] vs. 0.30 [0.18–0.43], *p* < 0.001). The preoperative–postoperative differences in RMT in the HCS subgroup were significantly lower than those in the ES subgroup (0.10 [0.06–0.15] vs. 0.30 [0.18–0.43], *p* < 0.001) (Table 4).

**TABLE 4:** Comparison of the treatment outcomes of different surgical procedures among Type 2 CSP patients.

	VAS	HCS	*p* value	VAS	ES	*p* value	HCS	ES	*p* value
Uterine diverticulum, *n* (%)									
Yes	197 (61.0)	351 (97.8)	< 0.001	197 (61.0)	7 (20.6)	< 0.001	351 (97.8)	7 (20.6)	< 0.001
No	126 (39.0)	8 (2.2)		126 (39.0)	27 (79.4)		8 (2.2)	27 (79.4)	
Value difference of RMT, (cm)									
Medians (IQRs)	0.11 (0.06–0.20)	0.10 (0.06–0.15)	0.011	0.11 (0.06–0.20)	0.30 (0.18–0.43)	< 0.001	0.10 (0.06–0.15)	0.30 (0.18–0.43)	< 0.001

## 4. Discussion

There have been numerous reports in the literature on the effectiveness of treatment modalities for CSP and the associated risks of recurrent CSP. However, research on the effect of selecting treatment approaches guided by different CSP types on the remaining cesarean scar diverticulum and RMT is lacking. RMT is an independent risk factor for CSP [21]. Both the occurrence of surgical complications and the recurrence of CSP are closely related to RMT. When the RMT is < 3 mm, hysteroscopic CSP resection should not be performed [8, 22]. Some scholars agree that laparotomic resection serves as the optimal treatment choice for CSP [12]. Ultrasound‐guided vacuum aspiration after the local injection of lauromacrogol combined with hysteroscopy should be selected depending on the gestational week [23]. This recommendation is based on efficacy and safety, particularly in cases where conservative treatment has failed, uterine rupture has occurred, or there is a suspicion of uterine rupture necessitating surgical intervention. Additionally, the decision to opt for CSP resection is influenced by the relatively high risks associated with alternative surgical approaches, as evidenced by postoperative evaluations [10]. The surgical outcomes and pregnancy outcomes associated with Type 1 CSP are generally favorable [24]. The surgical risk for Type 2 CSP is greater than that for Type 1 CSP; for example, in our study, there was a statistically significant difference in uterine artery embolization between the Type 2 CSP and Type 1 CSP groups, and the pregnancy sac diameter in the Type 2 CSP group was significantly greater than that in the Type 1 CSP group. However, there was no difference in the incidence of complications between the Type 1 CSP group and the Type 2 CSP group.

Conservative treatment approaches for CSP can terminate CSP pregnancies but are associated with an increased CSP recurrence rate [25]. In our study, compared with conservative surgeries, ES surgery reduced the detection rate of cesarean scar diverticulum. This may explain the correlation between persistent cesarean scar diverticulum and recurrent CSP. We agree with He et al. [26] that through hysteroscopic or laparoscopic resection of CSP, cesarean scar diverticulum can be effectively treated, serving as an effective means to improve the rate of subsequent pregnancies and reduce the recurrence rate of CSP. The potential mechanism accounting for the lower cesarean scar diverticulum detection rate and decreased risk of recurrent CSP after ES is summarized as follows: ES closes the diverticulum and resects scar tissue surgically, alleviating fibrosis and increasing cellularity within the neoendometrium. These changes promote decidualization and optimize the intrauterine microenvironment.

In our study, the detection rate of cesarean scar diverticulum significantly increased in Type 2 CSP patients after surgery, suggesting that cesarean scar diverticulum size may be positively correlated with the risk of CSP. The preoperative–postoperative differences of RMT in the Type 2 CSP group were greater than those in the Type 1 CSP group after surgery, suggesting that, under the guidance of the Chinese classification system, adjustments to the surgical approach, such as CSP excision and repair, when closing the uterine incision, effectively improved the RMT. The present study focuses on the anatomical remodeling of the uterine scar following various surgical procedures and further explores the guidance of the Chinese classification for CSP on surgical strategy selection. This study is expected to provide new evidence to support uterine scar remodeling and surgical approach selection as guided by the classification of CSP.

### 4.1. Limitations

There are several limitations in our study that should be acknowledged. First, our study was limited because the sample size of the resection group was relatively small, and the size of the gestational sac was not considered [27]. Second, although our study selected surgical approaches on the basis of propensity scores, selection bias remained regarding the choice of surgical approach. The choice of surgical approach may be influenced by confounding factors such as surgeon clinical skills, patient understanding of the surgical procedure, varying gestational ages, and unpredictable bleeding tendencies. Third, the study did not include a cost‐effectiveness analysis. The absence of a cost‐effectiveness evaluation leaves a critical gap in understanding the practical utility of the results.

## 5. Conclusion

ES helps reduce the incidence of uterine diverticulum and increase the thickness of the residual myometrium among patients with endogenous CSP.

## Author Contributions

Zhujie Wang: methodology, writing–original draft, and data curation. Xingmiao Li: writing–original draft. Ning Wang: data curation, software, and validation. Feng Qi: data curation, formal analysis, and resources. Jianwei Zhou: data curation. Meijun Guo: data curation and investigation. Mengjia Yu: data curation and investigation. Xuelu Zhu: data curation and investigation. Yanan Yang: investigation, validation, and visualization. Xiuxiu Jiang: conceptualization, funding acquisition, project administration, validation, and writing–review and editing.

## Funding

Funding was provided by the Zhejiang Provincial Central Leading Local Science and Technology Development Fund Project (2024ZY01028), the Zhejiang Province’s 2025 ‘Pioneer Leading Swan + X’ Science and Technology Program (2025C02122), and the 4+X Clinical Research of Women’s Hospital, School of Medicine, Zhejiang University (ZDFY2023‐4XPY101).

## Conflicts of Interest

The authors declare no conflicts of interest.

## Supporting Information

Additional supporting information can be found online in the Supporting Information section.

## Supporting information


**Supporting Information** Supporting 1. The recommended surgical approach model was matched based on the risk score for recurrent CSP. Supporting 2. Supporting information was originally published as part of our previous work based on the same cohort study [19, 20]. Supporting 3. Recommended surgical treatment options based on score ranges: ≤ 7 points (VAS), 8–13 points (HCS), and ≥ 14 points (ES). A risk scoring table was derived through regression analysis of the risks associated with recurrent CSP. The covariates included in the matching were patient age, type of CSP, gestational age, gestational sac size, fertility desire, and the number of previous CSP occurrences. Multivariable logistic regression analysis was conducted, and scores were assigned based on statistically significant variables. The predictive scores were calculated using a nomogram model for patients with recurrent CSP.

## Data Availability

The data that support the findings of this study are available on request from the corresponding author. The data are not publicly available due to privacy or ethical restrictions.
