# Supplementary material for: Treatment Outcomes of Excisional Versus Conservative Surgery for Endogenous Cesarean Scar Pregnancy: A Retrospective Cohort Study
Source: Obstet Gynecol Int. 2026 Sep 16;2026:5246199. doi: 10.1155/ogi/5246199 (PMC13579242; doi:10.1155/ogi/5246199)
Supplement: Supplementary file 1 [file OGI-2026-5246199-s001.zip › Supplementary material .docx]

**Supplementary material 2**

**The recommendation surgical approach model matched based on the risk score for recurrent CSP**

| **Risk factors** |  | **Score** |
| --- | --- | --- |
| **Gestational age (Week)** | **<8w** | **1** |
|  | **≥8w** | **2** |
| **Chinese classification** | **I** | **2** |
|  | **II** | **3** |
|  | **III** | **10** |
| **Gestational sac diameter（cm）** | **<3.5** | **1** |
|  | **≥3.5** | **3** |
| **Fertility desire** | **Yes** | **3** |
|  | **No** | **1** |
| **Number of previous CSP occurrences** | **0** | **1** |
|  | **1** | **6** |
|  | **≧2** | **10** |
| Note: Recommended surgical treatment options based on score ranges: ≤7 points (VAS), 8-13 points (HCS), ≥14 points (ES); A risk scoring table was derived through regression analysis of the risks associated with recurrent CSP, and predictive scores were calculated using a nomogram model for patients with recurrent CSP. | | |
