# Supplementary material for: Treatment Outcomes of Excisional Versus Conservative Surgery for Endogenous Cesarean Scar Pregnancy: A Retrospective Cohort Study
Source: Obstet Gynecol Int. 2026 Sep 16;2026:5246199. doi: 10.1155/ogi/5246199 (PMC13579242; doi:10.1155/ogi/5246199)
Supplement: Supplementary file 1 [file OGI-2026-5246199-s001.zip › Supplementary material_2.docx]

**Supplementary material**

**The recommendation surgical approach model matched based on the risk score for recurrent CSP**

| **Risk factors** |  | **Score** |
| --- | --- | --- |
| **Gestational age (week)** | <8w  ≥8w | 1  2 |
| **Chinese classification** | I  II  III | 2  3  10 |
| **Gestational sac diameter(cm)** | <3.5  ≥3.5 | 1  3 |
| **Fertility desire** | Yes  No | 3  1 |
| **Number of previous CSP occurrences** | 0  1  ≧2 | 1  6  10 |

Note: Recommended surgical treatment options based on score ranges: ≤7 points (VAS), 8-13 points (HCS), ≥14 points (ES); A risk scoring table was derived through regression analysis of the risks associated with recurrent CSP. The covariates included in the matching were patient age, type of CSP, gestational age, gestational sac size, fertility desire, and number of previous CSP occurrences. Multivariable logistic regression analysis was conducted, and scores were assigned based on statistically significant variables. The predictive scores were calculated using a nomogram model for patients with recurrent CSP.
